# Baseline RDW combined with dynamic trajectory: Predictive value for 30-day all-cause mortality in patients with sepsis-induced coagulopathy and development of a nomogram

**DOI:** 10.1371/journal.pone.0348149

**Published:** 2026-04-27

**Authors:** Ying Yang, Tianyang Chen, Qian Wang, Qian Chen

**Affiliations:** 1 Shanghai University of Traditional Chinese Medicine, Shanghai, China; 2 Emergency Department, Shuguang Hospital Affiliated to Shanghai University of Traditional Chinese Medicine, Shanghai, China; 3 Department of Critical Care Medicine, Henan Provincial Hospital of Traditional Chinese Medicine, Zhengzhou, China; University of Diyala College of Medicine, IRAQ

## Abstract

**Objective:**

Sepsis-induced coagulopathy (SIC) is associated with high mortality. This study aimed to explore the predictive value of baseline red blood cell distribution width (RDW) and its dynamic trajectory for 30-day all-cause mortality in SIC patients, and to develop a practical nomogram.

**Methods:**

A retrospective cohort study was conducted on 2531 SIC patients from the MIMIC-IV v3.1 database. Patients were grouped by baseline RDW tertiles, and RDW dynamic trajectories were constructed via Latent Class Growth Mixture Model (LCGMM). Kaplan-Meier analysis, Cox proportional hazards regression, and Restricted Cubic Spline (RCS) model were applied to assess the association between RDW and 30-day mortality. A nomogram was built via Boruta algorithm and Lasso regression, with external validation in 317 patients from a Shanghai tertiary hospital.

**Results:**

30-day mortality increased with elevated baseline RDW (Q1: 4.5% vs. Q2: 10.7% vs. Q3: 22.7%, *P* < 0.001), and Q3 was an independent risk factor (adjusted HR = 2.666, 95%CI: 1.854–3.834). RDW> about 15% correlated with sustained mortality risk. LCGMM identified two trajectories (stable low-level Traj0, rapidly ascending Traj1), with Traj1 showing higher mortality (31.1% vs. 10.0%, adjusted HR = 2.522). The nomogram integrating RDW and clinical indicators demonstrated good discrimination (C-index = 0.805, AUC = 0.813) and utility.

**Conclusion:**

High baseline RDW and rapidly ascending RDW trajectory are independent risk factors for 30-day mortality in SIC patients. The nomogram enables convenient and accurate risk stratification and prognostic evaluation.

## 1. Introduction

Sepsis is one of the leading causes of death in intensive care unit (ICU) patients. Its pathophysiological process is complex and often accompanied by coagulation dysfunction, namely sepsis-induced coagulopathy (SIC) [1]. Statistical data show that the prevalence of SIC in sepsis patients is approximately 22.1%−42.2%, and as high as 66.4% in patients with septic shock. Its occurrence is closely associated with higher mortality, morbidity, and poor clinical prognosis [2–4]. The SIC scoring criteria released by the International Society on Thrombosis and Haemostasis (ISTH) in 2019 (based on platelet count, international normalized ratio (INR), and the sum of Sequential Organ Failure Assessment (SOFA) subscores for the heart, liver, kidney and respiratory systems) [5] have been validated as a unified basis for clinical diagnosis. However, further accurate stratification of patients’ mortality risk, identification of populations with poor prognosis, and development of convenient and feasible clinical prediction tools remain key challenges in clinical practice.

In recent years, red blood cell distribution width (RDW), a routine laboratory indicator reflecting the heterogeneity of red blood cell size [6], has attracted attention in the prognostic evaluation of critical illnesses such as sepsis, myocardial infarction, and malignant tumors due to its convenient detection and low cost [7–11]. Existing studies have confirmed that elevated baseline RDW in sepsis patients is associated with aggravated organ dysfunction, prolonged hospital stay, and increased short-term mortality [12,13]. However, most current evidence focuses only on the predictive value of static baseline RDW, ignoring the dynamic evolution of RDW during the course of critical illnesses. This limitation makes it difficult to capture the dynamic changes of the disease, thereby affecting the accuracy of prognostic evaluation. In addition, for the special population with SIC, the association mechanism between RDW and coagulation dysfunction as well as organ injury remains unclear: on the one hand, elevated RDW may indirectly enhance coagulation system activation and microthrombus formation by reflecting abnormal erythropoiesis and increased oxidative stress [6,14–16]; on the other hand, liver and kidney function damage and inflammatory factor storms often accompanied by SIC may reversely affect red blood cell metabolism, leading to dynamic changes in RDW [1,6,17].

At present, there is a lack of research on the predictive value of baseline RDW level and dynamic trajectory for 30-day all-cause mortality in SIC patients, and no study has developed a prognostic prediction model for SIC patients based on RDW. Based on this, this study retrospectively analyzed the clinical data of SIC patients in the MIMIC-IV v3.1 database to clarify the association and dose-response relationship between baseline RDW and 30-day all-cause mortality in patients; constructed RDW dynamic trajectories using the Latent Class Growth Mixture Model (LCGMM) to explore its prognostic value; simultaneously verified subgroup robustness and generalization ability in an independent external cohort; and finally developed a nomogram prediction model based on core predictive variables, aiming to provide new biomarkers and convenient clinical prediction tools for SIC risk stratification.

## 2. Methods

### 2.1. Data source

This was an observational cohort study. The main data were derived from the Information Mart for Intensive Care IV (MIMIC-IV) v3.1 database [18], which was developed by the Laboratory for Computational Physiology at the Massachusetts Institute of Technology (MIT) and contains comprehensive clinical data of ICU patients at the Beth Israel Deaconess Medical Center (BIDMC) from 01/01/2008–31/12/2019. Secondary use of the data has been approved by the Institutional Review Boards of BIDMC and MIT (Certification No.: 68349734). In addition, the external validation data of this study were derived from SIC patients admitted to the ICU of a Grade A tertiary hospital in Shanghai from 12/05/2022–11/05/2023. The relevant study has been approved by the Medical Ethics Committee of the hospital (Ethics No.: 2021-971-46-02). Since this study was a retrospective analysis and all data had been de-identified, which met the requirements of ethical review, informed consent of patients was waived.

### 2.2. Study design and study population

**Fig 1:**
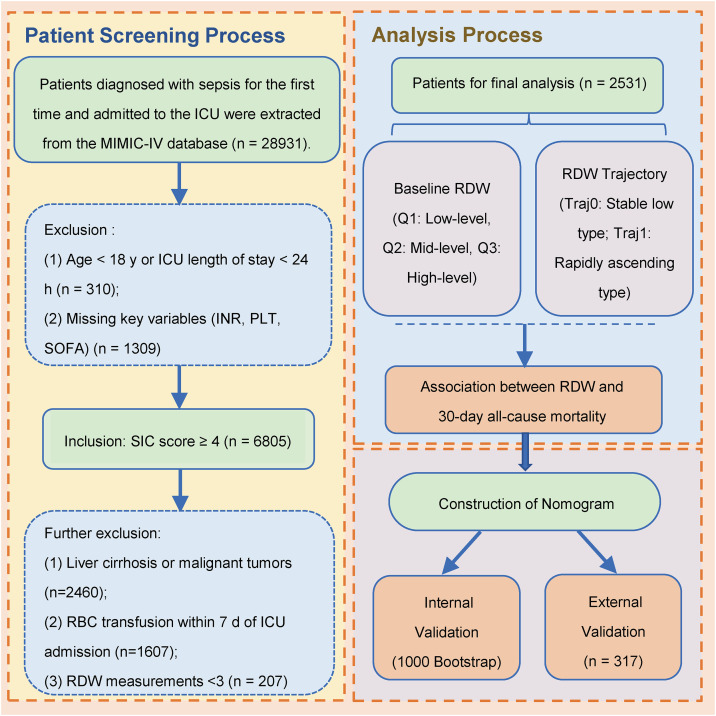
Flowchart of patient screening and analysis.

According to the Sepsis 3.0 definition (SOFA score ≥ 2 accompanied by infection) [19], sepsis patients admitted to the ICU were extracted. According to the 2019 ISTH SIC scoring criteria, SIC was diagnosed if the sum of any two of the three indicators (platelet count, INR, and the sum of SOFA subscores for heart, liver, kidney and respiratory systems) was ≥ 4 points. All SIC patients were screened from MIMIC-IV. Meanwhile, the following patients were excluded: (1) Age < 18 years or ICU length of stay < 24 hours; (2) Number of RDW detections during ICU stay < 3 times; (3) Missing key variables (INR, PLT, SOFA) on the first day of ICU admission, making it impossible to diagnose coagulopathy; (4) Complicated with liver cirrhosis or malignant tumors; (5) Received red blood cell transfusion within 7 days of ICU admission [20]. For patients admitted to the ICU multiple times, only the data of the first admission were included. Finally, 2531 patients met all inclusion and exclusion criteria. The study flow is shown in Fig 1.

### 2.3. Data extraction and processing

#### 2.3.1. Data extraction.

Data within 24 hours after ICU admission were extracted from the MIMIC-IV database using Structured Query Language (SQL) with DecisionLinnc 1.0 software. Variables were divided into six categories: (1) Demographic information: age, gender; (2) Comorbidities: diabetes mellitus (DM), hypertension (HTN), chronic kidney disease (CKD); (3) Laboratory indicators: The baseline values of all laboratory indicators were the first test results within 24 hours after ICU admission, including total bilirubin (TBIL), albumin (ALB), international normalized ratio (INR), platelet count (PLT), red blood cell distribution width (RDW), C-reactive protein (CRP), lactate (Lac), hemoglobin (Hb), hematocrit (HCT), red blood cell (RBC), ferritin, transferrin, activated partial thromboplastin time (APTT), D-dimer, functional fibrinogen, folic acid, vitamin B12, creatinine (Cr); (4) Interventions: The use of interventions within 24 hours after ICU admission was extracted, including mechanical ventilation (MV), continuous renal replacement therapy (CRRT), vasopressors (VP), fresh frozen plasma (FFP), therapeutic heparin; (5) Disease severity scores: Sequential Organ Failure Assessment (SOFA) score, Charlson Comorbidity Index; (6) Repeated RDW test data within 7 days: The time range was from Day 1 to Day 7 after ICU admission, excluding measurements within the first 24 hours. To reduce the impact of random fluctuations of a single test on the trajectory shape, the average value of multiple tests on the same day was used to represent the daily RDW level.

#### 2.3.2. Data processing.

For non-core variables with a missing rate < 30%, multiple imputation was used for data filling [21]; variables with a missing rate > 30% were excluded. The missing status of variables is shown in S1 Table in S1 File. Multiple imputation was performed under a Markov Chain Monte Carlo (MCMC) framework. Predictive mean matching (PMM) was applied as the imputation algorithm to preserve the original distribution and correlations of variables. A total of 5 imputed datasets were generated to account for uncertainty in missing values. Convergence of the imputation process was assessed using trace plots, and distributions of observed and imputed values were compared to ensure the reliability of the imputation procedure. The imputation model included all variables in the final Cox model and other clinically relevant covariates with missing rates < 30%. In addition, variance inflation factor (VIF) collinearity test was performed on variables included in the multivariable regression model (S2 Table in S1 File) [22]. The results showed that the VIF values of Hb and HCT were both > 5, indicating a certain degree of collinearity. Combined with clinical relevance and the preliminary association strength between variables and outcomes, Hb and HCT were finally excluded, and only RBC was retained for subsequent analysis.

### 2.4. Outcome indicators and variable definitions

The primary outcome indicator was 30-day all-cause mortality. Definitions of key variables: (1) Baseline red blood cell distribution width (RDW) (first test value) tertiles: low-level (Q1: < 13.7%), mid-level (Q2: 13.7%−15.3%), high-level (Q3: > 15.3%); (2) RDW dynamic trajectory: divided into stable low-level type (Traj0) and rapidly ascending type (Traj1) by the LCGMM.

### 2.5. Statistical analysis

Continuous variables were non-normally distributed (Shapiro-Wilk test) and expressed as median (interquartile range, IQR); categorical variables were expressed as count (percentage).

#### 2.5.1. Baseline RDW and 30-day mortality analysis.

To explore the association between baseline RDW (measured within 24 hours of ICU admission) and SIC prognosis, we used the Kaplan-Meier (KM) method to generate 30-day survival curves and the log-rank test to compare mortality across the three groups; we then used Cox proportional hazards regression models to evaluate the independent predictive value of baseline RDW for 30-day mortality. Three models were constructed to adjust for confounding factors: Model 1: Only core indicators (baseline RDW tertiles) were included without adjustment for other covariates; Model 2: Age and gender were added on the basis of Model 1; Model 3: SOFA score, red blood cell count (RBC), treatment factors (therapeutic heparin, MV, CRRT, VP, FFP), and comorbidities (DM, HTN, CKD) were further adjusted on the basis of Model 2. We constructed a directed acyclic graph (DAG) to clarify the causal relationships between exposure, covariates, and outcome (S1 Fig in S1 File). Baseline illness severity was identified as the common ancestor of both early interventions and subsequent RDW trajectory changes, confirming interventions as baseline confounders rather than mediators or colliders. The proportional hazards assumption was tested using Schoenfeld residuals. The global test for each model was not significant (all *P* > 0.05), indicating no violation of the proportional hazards assumption. We used the Restricted Cubic Spline (RCS) model (based on the confounding factors adjusted in Model 3) to test the non-linear association between baseline RDW and mortality risk.

#### 2.5.2. RDW dynamic trajectory construction by LCGMM.

Based on the daily average RDW from Day 1 to Day 7 after ICU admission, LCGMM was used to fit the dynamic trajectory. LCGMM is a grouping method based on longitudinal data that identifies potential subgroups with similar dynamic trajectories [23]. The model assumes that the parameters of RDW change curves of individuals in the same potential class are consistent, and all subjects are randomly selected from latent classes with heterogeneous development trajectories. The trajectory trend of each potential class is modeled using a linear polynomial function; this choice was made for its alignment with the typical clinical evolution of RDW in SIC patients within the first 7 days after ICU admission, favorable clinical interpretability, and ability to mitigate overfitting risks associated with higher-order polynomial functions in our dataset. Model convergence was verified by the convergence (Conv) indicator, where a value of 1 denotes successful algorithm convergence; all tested models with 2–5 trajectory classes achieved convergence (Conv = 1), ensuring the stability and reliability of parameter estimation. During the fitting process, the posterior probability of each individual belonging to each trajectory class is estimated synchronously, and individuals are assigned to the corresponding subgroups according to the highest posterior probability [24,25]. Finally, the optimal trajectory class was determined comprehensively based on information criteria (AIC, SABIC), classification reliability (average posterior probability ≥ 0.7), entropy value, minimum class proportion (≥ 5%), and clinical rationality [26].

#### 2.5.3. RDW trajectory and 30-day mortality analysis.

KM method was used to draw 30-day survival curves of different RDW trajectory groups, and log-rank test was used to compare the mortality differences among groups; the three stepwise adjusted Cox proportional hazards regression models mentioned above were used to verify the independent association between RDW dynamic trajectory and 30-day mortality.

#### 2.5.4. Subgroup and interaction analysis.

To further verify the robustness of the results, subgroup analysis was carried out by stratifying according to age (≥ 65 years vs. < 65 years), Hb (< 10 g/L vs. ≥ 10 g/L), INR (< 1.5 vs. ≥ 1.5), DM, HTN, CKD, VP use, MV use, CRRT use, and FFP use. The prognostic association of RDW trajectory was verified in each subgroup and interaction was tested. Bonferroni method was used to adjust the *P* value of each subgroup.

#### 2.5.5. Sensitivity analysis.

To address potential survivor bias in trajectory modeling and prognostic analysis, a sensitivity analysis was performed. Specifically, patients who died within 72 hours after ICU admission were excluded, and the entire analytical pipeline (including LCGMM trajectory identification and multivariable Cox proportional hazards regression) was repeated in the remaining cohort to validate the robustness of the primary findings.

#### 2.5.6. Incremental prognostic value.

The incremental prognostic value of RDW dynamic trajectory was evaluated by comparing nested models: a clinical baseline model, a model incorporating baseline RDW, and a model further integrating RDW dynamic trajectory. Model fitting efficacy was assessed via likelihood ratio tests, while predictive performance was compared using C-index and area under the receiver operating characteristic curve (AUC), with statistical testing performed to quantify changes in discriminative ability.

### 2.6. Nomogram construction and validation

To screen core variables for nomogram construction, Boruta algorithm combined with Lasso regression was used [27]. First, the Boruta algorithm was used to compare the importance of real variables and shadow variables for identifying key features. Subsequently, Lasso regression was applied to retain variables with non-zero coefficients through L1 regularization shrinkage, where the lambda value was determined based on the minimum mean squared error from 10-fold cross-validation combined with the one-standard-error rule. Finally, the variables were used for nomogram construction. C-index, time-dependent ROC curve (AUC), calibration curve, and decision curve analysis (DCA) were used to evaluate the discrimination, calibration, and clinical utility of the model; 1000 Bootstrap resampling was used for internal validation to test the model stability.

The external validation cohort included SIC patients admitted to the ICU of a Grade A tertiary hospital in Shanghai from May 2022 to May 2023. Strictly following the inclusion and exclusion criteria of the internal cohort, 317 patients were finally included. This cohort was selected to independently verify the model generalization ability, and the same evaluation indicators as the training set were used.

All analyses were performed using DecisionLinnc 1.0 software [28], and a two-sided *P* < 0.05 was considered statistically significant.

## 3. Results

### 3.1. Baseline characteristics of the study population

A total of 2531 patients with sepsis-induced coagulopathy were included in this study. According to the baseline red blood cell distribution width (RDW) tertiles, they were divided into low-level group (Q1: < 13.7%, n = 846), mid-level group (Q2: 13.7%−15.3%, n = 847), and high-level group (Q3: > 15.3%, n = 838). As shown in Table 1, there were significant differences in baseline characteristics among the three groups. The Q3 group had more severe conditions: patients in this group were older (median 72.0 years) with a higher Charlson Comorbidity Index (6.0). Although the median SOFA score was consistent with the other two groups, the heterogeneity reflecting the severity of organ injury was greater; the creatinine was higher (1.6 mg/dL), indicating more severe coagulation dysfunction, renal insufficiency, and hypoxemia; at the same time, the CRRT utilization rate (4.1%), diabetes prevalence (40.2%), and chronic kidney disease prevalence (33.7%) were higher than those in the other two groups.

**Table 1:** Baseline characteristics of patients with sepsis-induced coagulopathy stratified by baseline RDW tertiles.

	Overall (N = 2531)	Q1: Low-level (n = 846)	Q2: Mid-level (n = 847)	Q3: High-level (n = 838)	*P*
**Demographics**					
Age (years)	69.0 (21.0)	66.0 (18.0)	71.0 (22.0)	72.0 (20.0)	<0.001
Gender (Male, n%)	1666 (65.8)	630 (74.5)	533 (62.9)	503 (60.0)	<0.001
**Disease Severity Scores**					
SOFA	7.0 (4.0)	7.0 (4.0)	7.0 (4.0)	7.0 (5.0)	<0.001
Charlson	5.0 (4.0)	3.0 (3.0)	5.0 (4.0)	6.0 (4.0)	<0.001
**Laboratory Markers**					
RBC (×10¹²/L)	3.4 (0.9)	3.4 (0.8)	3.5 (1.0)	3.4 (1.1)	0.001
INR	1.6 (0.4)	1.5 (0.3)	1.6 (0.4)	1.7 (0.7)	<0.001
APTT (s)	33.1 (10.5)	31.4 (8.5)	33.0 (9.7)	35.3 (12.8)	<0.001
PLT (×10⁹/L)	129.0 (80.0)	124.0 (51.0)	127.0 (73.0)	137.5 (125.0)	<0.001
Lac (mmol/L)	2.0 (1.6)	2.1 (1.4)	2.0 (1.5)	2.0 (1.7)	0.535
TBIL (mg/dL)	0.8 (1.2)	0.8 (1.0)	0.8 (1.0)	1.0 (1.7)	<0.001
Cr (mg/dL)	1.2 (1.1)	0.9 (0.5)	1.2 (1.0)	1.6 (1.7)	<0.001
**First-day Treatments**					
Therapeutic heparin (n%)	233 (9.2)	48 (5.7)	82 (9.7)	103 (12.3)	<0.001
MV (n%)	2052 (81.1)	739 (87.4)	680 (80.3)	633 (75.5)	<0.001
CRRT (n%)	62 (2.4)	5 (0.6)	23 (2.7)	34 (4.1)	<0.001
VP (n%)	1373 (54.2)	620 (73.3)	438 (51.7)	315 (37.6)	<0.001
FFP (n%)	123 (4.9)	32 (3.8)	47 (5.5)	44 (5.3)	0.195
**Comorbidities**					
DM (n%)	824 (32.6)	209 (24.7)	278 (32.8)	337 (40.2)	<0.001
HTN (n%)	989 (39.1)	420 (49.6)	331 (39.1)	238 (28.4)	<0.001
CKD (n%)	565 (22.3)	87 (10.3)	196 (23.1)	282 (33.7)	<0.001
**Clinical Outcome**					
30-day mortality (n%)	319 (12.6)	38 (4.5)	91 (10.7)	190 (22.7)	<0.001

Low-level: < 13.7%; Mid-level: 13.7%−15.3%; High-level: > 15.3%. Continuous variables are presented as median (IQR), and categorical variables are expressed as n (%).

RDW: Red blood cell distribution width; SOFA: Sequential Organ Failure Assessment; RBC: Red blood cell; INR: International normalized ratio; APTT: Activated partial thromboplastin time; PLT: Platelet; Lac: Lactate; TBIL: Total bilirubin; Cr: Creatinine; MV: Mechanical Ventilation; CRRT: Continuous renal replacement therapy; VP: Vasopressors; FFP: Fresh frozen plasma; DM: Diabetes mellitus; HTN: Hypertension; CKD: Chronic kidney disease.

The 30-day mortality of the three groups showed a gradual increase (Q1: 4.5% vs. Q2: 10.7% vs. Q3: 22.7%, *P* < 0.001), which further suggested that high baseline RDW was closely associated with poor prognosis of SIC patients. It is worth noting that the prevalence of hypertension in the Q3 group (28.4%) was lower than that in the Q1 group (49.6%) and Q2 group (39.1%), which may be related to the differences in the management status of underlying diseases and body reserve function in this subgroup [29]. It may also be attributed to inherent biases of observational studies (such as selection bias, information bias, or survival bias) that could affect the distribution of comorbidity data across groups; while the utilization rates of vasopressors (37.6%) and MV (75.5%) in the Q3 group were lower than those in the Q1 and Q2 groups. This contradictory phenomenon may be related to various factors, including the ceiling effect of treatment caused by more severe baseline status and complex comorbidities, or the inability to upgrade treatment due to early clinical deterioration [30,31].

### 3.2. Association between baseline RDW and 30-day mortality in SIC patients

#### 3.2.1. Survival differences in SIC patients stratified by baseline RDW.

**Fig 2:**
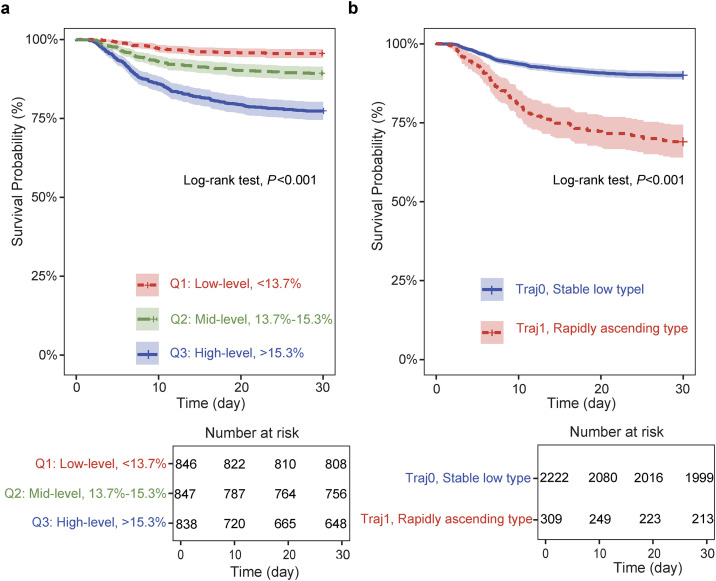
Kaplan-Meier curves for 30-day mortality. **(a)** Baseline RDW; **(b)** RDW Trajectory.

Kaplan-Meier survival curve analysis showed that the Q3 group (high RDW level) had the lowest survival probability, and the Q1 group (low RDW level) had the highest survival probability. The difference in survival probability among the three groups was statistically significant (*P* < 0.001), indicating that RDW can be used as an effective stratification indicator for short-term mortality risk in SIC patients. The Kaplan-Meier curve is shown in Fig 2.

#### 3.2.2. Independent association between baseline RDW and 30-day mortality.

To clarify the independent impact of baseline RDW on mortality, three stepwise adjusted Cox proportional hazards models were used for analysis (results are shown in Table 2): Model 1 only included baseline RDW tertiles (unadjusted); Model 2 adjusted for age and gender; Model 3 further adjusted for SOFA score, red blood cell count (RBC), treatment factors (therapeutic heparin, MV, CRRT, VP, FFP), and comorbidities (DM, HTN, CKD). Taking the low baseline RDW group as the reference, in Model 1, the mortality risk of the Q2 group (HR = 2.478, 95% CI: 1.697–3.619, *P* < 0.001) and Q3 group (HR = 5.595, 95% CI: 3.949–7.927, *P* < 0.001) was significantly increased; in Model 2, the HR of the two groups was 2.254 (95% CI: 1.539–3.300, *P* < 0.001) and 5.032 (95% CI: 3.537–7.159, *P* < 0.001) respectively; in Model 3, the association of the Q2 group was weakened to marginally significant (HR = 1.442, 95% CI: 0.981–2.121, *P* = 0.063), while the Q3 group still significantly increased the mortality risk (HR = 2.666, 95% CI: 1.854–3.834, *P* < 0.001). These results indicate that high baseline RDW is an independent risk factor for 30-day mortality in SIC patients, and this association remains stable after adjusting for confounding factors.

**Table 2:** Association of baseline RDW tertiles and RDW trajectories with 30-day all-cause mortality.

Variables	Model 1		Model 2		Model 3	
	HR (95% CI)	*P*	HR (95% CI)	*P*	HR (95% CI)	*P*
**Baseline RDW**						
Q1 (Low)	1.00 (Ref)		1.00 (Ref)		1.00 (Ref)	
Q2 (Medium)	2.478 (1.697–3.619)	<0.001	2.254 (1.539–3.300)	<0.001	1.442 (0.981–2.121)	0.063
Q3 (High)	5.595 (3.949–7.927)	<0.001	5.032 (3.537–7.159)	<0.001	2.666 (1.854–3.834)	<0.001
**RDW Trajectory**						
Traj0	1.00 (Ref)		1.00 (Ref)		1.00 (Ref)	
Traj1	3.466 (2.728–4.403)	<0.001	3.876 (3.042–4.940)	<0.001	2.522 (1.964–3.238)	<0.001

Model 1: Unadjusted;

Model 2: Adjusted for age and gender;

Model 3: Adjusted for Model 2 + SOFA, RBC, heparin, FFP, MV, CRRT, VP, DM, HTN, CKD.

#### 3.2.3. Dose-response relationship between baseline RDW and 30-day mortality.

To clarify the association pattern between red blood cell distribution width (RDW) and outcome risk, this study further analyzed using the Restricted Cubic Spline (RCS) model. The results showed (S2 Fig in S1 File) that RDW had an approximately linear association with hazard ratio (HR) (non-linear test *P* = 0.099): when RDW was lower than about 15%, HR was slightly lower than 1, indicating that RDW within this range had no significant impact on outcome risk; while when RDW exceeded about 15%, HR showed a continuous upward trend with the increase of RDW level, indicating that the higher the RDW, the significantly increased mortality risk. The overall association test *P* < 0.001 further suggested that there was a significant statistical association between RDW and outcome risk.

### 3.3. Construction of RDW dynamic trajectories and comparison of intergroup characteristics in SIC patients

#### 3.3.1. Determination of the optimal class of RDW dynamic trajectories.

The aforementioned analysis has clarified the linear association between baseline RDW level and patient outcome risk. To further reveal the dynamic evolution law and clinical significance of RDW, this study constructed patient RDW dynamic trajectories using LCGMM and determined the optimal number of groups.

**Fig 3:**
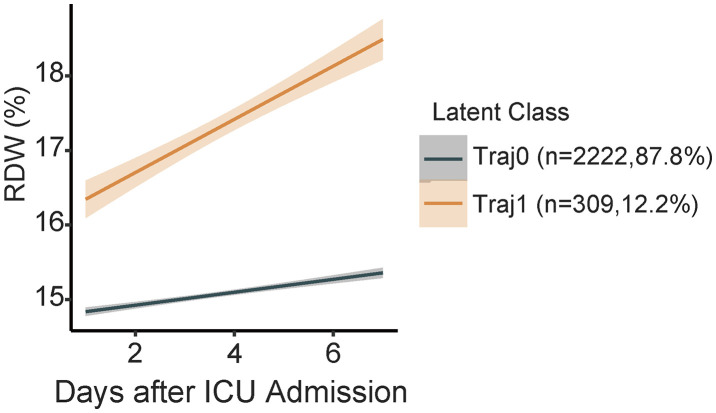
Classification of RDW trajectories in SIC patients (LCGMM, Day 1-7 after ICU admission).

According to the preset selection criteria, the 2-class model was finally determined as the optimal model: its AIC (28619.8051 vs. 31942.1185) and BIC (28666.4960 vs. 31965.4640) were significantly lower than those of the 1-class model, with a better fitting effect; the average posterior probabilities of the two classes both exceeded 90% (Class 1: 97.58%, Class 2: 92.53%), and 91.26% of individuals had a posterior probability ≥0.8 for their class, among which 96.49% of individuals in Class 1 had a posterior probability > 0.8, and 85.11% in Class 2. Combined with the entropy value of 0.8822, it indicated that the classification result was reliable; at the same time, there were no redundant small classes (Class 1: n = 2222, 87.8%, “stable low-level type”; Class 2: n = 309, 12.2%, “rapidly ascending type”), and the difference between the two trajectories had clear clinical significance (Fig 3), which was easy to interpret and could support subsequent analysis. The comparison of each model is shown in Table 3.

**Table 3:** Statistics for choosing the best number of RDW trajectories.

G	Conv	AIC	BIC	SABIC	entropy	PP1	PP2	PP3	PP4	PP5
1	1	31942.1185	31965.4640	31952.7549	1	100	–	–	–	–
2*	1	28619.8051	28666.4960	28641.0779	0.8822	87.7914	12.2086	–	–	–
3	1	27440.2835	27510.3199	27472.1927	0.8114	9.6405	79.3757	10.9838	–	–
4	1	26124.2031	26217.5850	26166.7488	0.8366	2.0545	16.2782	73.8048	7.8625	–
5	1	25773.1261	25889.8535	25826.3082	0.7168	28.4473	1.5014	52.9040	13.8285	3.3188

*The optimal trajectory model (determined by AIC, SABIC, entropy, classification reliability, minimum class proportion, and clinical rationality).

G: Growth trajectories number; Conv: Convergence; AIC: Akaike Information Criterion; BIC: Bayesian information criteria; SABIC: Sample-adjusted bayesian information criterion; PP: posterior probability.

#### 3.3.2. Comparison of baseline characteristics between different RDW dynamic trajectory groups.

To clarify the differences between RDW dynamic trajectory groups, the baseline characteristics of Traj1 (rapidly ascending type, n = 309) and Traj0 (stable low-level type, n = 2222) were compared (Table 4). The clinical condition of the Traj1 group was more severe: the median SOFA score (8.0 vs. 7.0) and Charlson Comorbidity Index (6.0 vs. 5.0) of the Traj1 group were significantly higher (both *P* < 0.001); laboratory indicators showed more obvious physiological disorders in the Traj1 group, with higher levels of lactate (2.5 vs. 2.0 mmol/L), total bilirubin (1.2 vs. 0.8 mg/dL), and creatinine (1.4 vs. 1.1 mg/dL) (all *P* < 0.001); in terms of interventions, the utilization rate of continuous renal replacement therapy (CRRT) in the Traj1 group was significantly higher (6.1% vs. 1.9%, *P* < 0.001), but the prevalence of hypertension was lower (28.5% vs. 40.5%, *P* < 0.001). Therefore, the 30-day mortality of the Traj1 group was significantly higher than that of the Traj0 group (31.1% vs. 10.0%, *P* < 0.001), highlighting the strong association between the rapidly ascending RDW dynamic trajectory and poor prognosis.

**Table 4:** Baseline characteristics of patients with sepsis-induced coagulopathy stratified by RDW trajectory groups.

	Overall (N = 2531)	Traj0 (n = 2222)	Traj1 (n = 309)	*P*
**Demographics**				
Age (years)	69.0 (21.0)	69.0 (20.0)	68.0 (20.0)	0.018
Gender (Male, n%)	1,666 (65.8)	1,500 (67.5)	166 (53.7)	<0.001
**Disease Severity Scores**				
SOFA	7.0 (4.0)	7.0 (4.0)	8.0 (5.0)	<0.001
Charlson	5.0 (4.0)	5.0 (4.0)	6.0 (5.0)	<0.001
**Laboratory Markers**				
RBC (×10¹²/L)	3.4 (0.9)	3.4 (0.9)	3.4 (1.1)	0.285
INR	1.6 (0.4)	1.6 (0.5)	1.7 (0.8)	<0.001
APTT (s)	33.1 (10.5)	32.9 (9.9)	34.8 (12.4)	<0.001
PLT (×10⁹/L)	129.0 (80.0)	129.0 (78.0)	131.0 (118.0)	0.893
Lac (mmol/L)	2.0 (1.6)	2.0 (1.5)	2.5 (2.4)	<0.001
TBIL (mg/dL)	0.8 (1.2)	0.8 (1.1)	1.2 (2.0)	<0.001
Cr (mg/dL)	1.2 (1.1)	1.1 (1.0)	1.4 (1.6)	<0.001
**First-day Treatments**				
Therapeutic heparin (n%)	233 (9.2)	198 (8.9)	35 (11.3)	0.169
MV (n%)	2052 (81.1)	1821 (82.0)	231 (74.8)	0.002
CRRT (n%)	62 (2.4)	43 (1.9)	19 (6.1)	<0.001
VP (n%)	1373 (54.2)	1280 (57.6)	93 (30.1)	<0.001
FFP (n%)	123 (4.9)	102 (4.6)	21 (6.8)	0.091
**Comorbidities**				
DM (n%)	824 (32.6)	711 (32.0)	113 (36.6)	0.108
HTN (n%)	989 (39.1)	901 (40.5)	88 (28.5)	<0.001
CKD (n%)	565 (22.3)	475 (21.4)	90 (29.1)	0.002
**Clinical Outcome**				
30-day mortality (n%)	319 (12.6)	223 (10.0)	96 (31.1)	<0.001

Traj0 (n = 2222, 87.8%): Stable low type; Traj1 (n = 309, 12.2%): Rapidly ascending type.

### 3.4. Association between RDW dynamic trajectory and 30-day mortality in SIC patients

#### 3.4.1. Survival differences in subgroups with different RDW dynamic trajectories in SIC patients.

Kaplan-Meier survival analysis showed that there were differences in 30-day mortality among different RDW dynamic trajectory groups: taking the Traj0 group as the reference, the survival probability of the Traj1 group decreased more significantly with time, and the two survival curves were clearly separated. Statistical test results showed that the difference between the two groups was highly statistically significant (*P* < 0.001), indicating that the 30-day mortality of patients in the Traj1 group was significantly higher than that in the Traj0 group, which suggested the potential predictive value of RDW dynamic trajectory for the short-term prognosis of SIC patients (Fig 2).

#### 3.4.2. Independent association between RDW dynamic trajectory and 30-day mortality.

To clarify the independent impact of RDW dynamic trajectory on mortality, the three stepwise adjusted Cox proportional hazards models mentioned above were used for analysis (results are shown in Table 2). Taking the Traj0 group as the reference, the mortality risk of the Traj1 group was significantly increased in Model 1 (HR = 3.466, 95% CI: 2.728–4.403, *P* < 0.001); HR = 3.876 in Model 2 (95% CI: 3.042–4.940, *P* < 0.001); the mortality risk decreased but was still statistically significant in Model 3 (HR = 2.522, 95% CI: 1.964–3.238, *P* < 0.001). This result was consistent with the trend of Cox analysis of baseline RDW, indicating that both static high level (Q3) and dynamic rapid increase (Traj1) were associated with increased mortality risk. Although the HR value of the Traj1 group (2.522) in Model 3 was slightly lower than that of the Q3 group (2.666), the 30-day mortality of the Traj1 group (31.1%) was higher, and it could capture the dynamic changes of the disease, maintaining a significant association in each model, suggesting that the rapid increase of RDW is an independent and relatively stable risk factor for 30-day mortality in SIC patients.

#### 3.4.3. Subgroup robustness analysis.

To further evaluate the robustness of the association between RDW dynamic trajectory and 30-day mortality under different clinical conditions, the interaction between RDW dynamic trajectory and age (≥ 65 years vs. < 65 years), Hb (< 10 g/L vs. ≥ 10 g/L), INR (< 1.5 vs. ≥ 1.5), DM, HTN, CKD, VP use, MV use, CRRT use, and FFP use was tested. The results of subgroup analysis are shown in S3 Fig in S1 File: in subgroups such as mechanical ventilation, continuous renal replacement therapy, hypertension, diabetes, vasopressors, INR, and fresh frozen plasma, the mortality risk of the rapidly ascending RDW group (Traj1) was significantly higher than that of the stable low-level group (Traj0) (*P* < 0.05); while in subgroups such as age, chronic kidney disease, and Hb, although the mortality risk of the Traj1 group showed an increasing trend, the difference in some subgroups did not reach statistical significance (*P* > 0.05), which may be related to the limited statistical power of smaller subgroups and residual confounding. Future studies need to further explore by expanding the sample size or more refined stratification analysis. At the same time, there was no statistical significance in the interaction between each subgroup and RDW dynamic trajectory (all *P* for interaction > 0.05), indicating that the predictive effect of RDW dynamic trajectory on mortality risk has good stability in most clinical subgroups, which further suggests its potential reliability as a prognostic biomarker for SIC patients.

#### 3.4.4. Sensitivity analysis.

To assess the potential impact of survivor bias on trajectory modeling and prognostic analysis, we performed a sensitivity analysis by excluding 31 patients who died within 72 hours after ICU admission, leaving 2500 eligible patients for reanalysis. Latent class growth mixture modeling (LCGMM) still identified an optimal 2-class solution (AIC = 28377.54, BIC = 28424.17, SABIC = 28398.75) with high classification reliability (entropy = 0.88622), and the trajectory distribution remained highly consistent with the original analysis (stable low-level trajectory: 87.90%; rapidly ascending trajectory: 12.10%). Multivariable Cox regression further confirmed that the rapidly ascending trajectory remained a robust independent risk factor for 30-day all-cause mortality (adjusted HR = 2.645, 95%CI: 2.381–2.933, *P* < 0.001), with an effect size and statistical significance nearly identical to the original result (adjusted HR = 2.522, 95%CI: 1.964–3.238, *P* < 0.001), indicating that survivor bias did not materially affect the core conclusions of this study.

### 3.5. Incremental prognostic value of RDW dynamic trajectory

We first compared the base clinical model (Model 1) with the model adding baseline RDW (Model 2). The likelihood ratio test showed that Model 2 fit significantly better (χ² = 56.08, df = 1, *P* < 0.001), with the C-index increasing from 0.7861 to 0.7998. The AUC was improved from 0.797 (Model 1) to 0.811 (Model 2) (S4 Fig in S1 File).

Although the C-index and AUC were comparable between Model 2 and Model 3 (C-index: 0.7998 vs 0.7983; AUC: 0.811 vs 0.812) (S4 Fig in S1 File), Model 3 still showed significantly better fit (χ² = 8.58, P = 0.0034) and higher log-likelihood. These results indicate that both baseline RDW and RDW dynamic trajectory provide independent prognostic information. While the C-index and AUC were comparable between Model 2 and Model 3, the significant improvement in model fit (likelihood ratio test) demonstrates that RDW dynamic trajectory captures meaningful temporal changes beyond a single baseline measurement, thereby enhancing prognostic stratification beyond baseline RDW alone.

### 3.6. Development and validation of the nomogram

#### 3.6.1. Variable screening.

**Fig 4:**
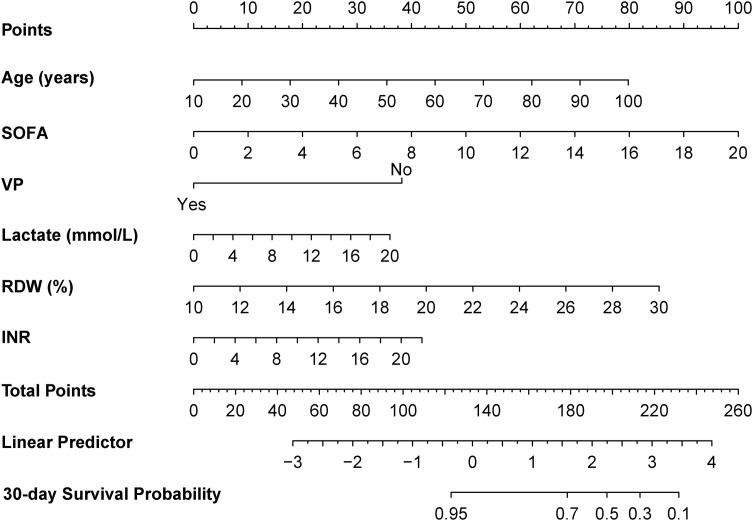
Nomogram for predicting 30-day mortality risk in ICU-admitted patients with SIC. For each patient, locate the value of each variable on the corresponding axis, draw a vertical line upward to obtain the respective points, sum the points of all variables to get the total points, and then draw a vertical line downward from the total points axis to the “30-day Survival Probability” axis to obtain the predicted 30-day survival probability of the SIC patient. Higher total points indicate a higher risk of 30-day all-cause mortality.

To screen core variables for nomogram construction, Boruta algorithm combined with Lasso regression was used. It can be seen that RDW successfully passed the strict screening of both algorithms, further suggesting its potential important predictive value for 30-day all-cause mortality in SIC patients (S5 Fig in S1 File). Finally, integrating the screening results, variables with non-zero coefficients and significant importance such as age, Sequential Organ Failure Assessment (SOFA) score, lactate (Lac), vasopressors (VP), international normalized ratio (INR), and RDW were retained to construct a nomogram prediction model (Fig 4). The finally retained core variables and their coefficients are detailed in S3 Table in S1 File.

#### 3.6.2. Model performance evaluation and internal validation.

**Fig 5:**
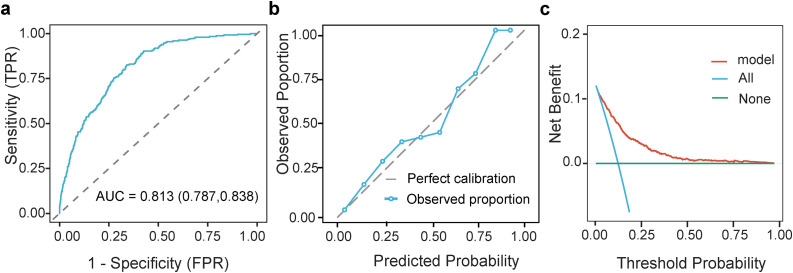
Performance of the nomogram in the training set. **A.** ROC Curve; **B.** Calibration Curve; **C.** Decision Curve Analysis.

Analysis of the training set showed that the C-index of the model was 0.805, and the AUC of the ROC curve was 0.813 (95% CI: 0.787–0.838); the 30-day calibration curve was close to the 45° diagonal, indicating that the predicted probability was well consistent with the actual probability, and the 30-day Brier score of the training set was 0.0887; the DCA curve had a higher net benefit than the “treat all” and “treat none” strategies when the threshold probability was < 0.5 (Fig 5). After 1000 Bootstrap resampling internal validation, the AUC was 0.813 (95% CI: 0.787–0.838), and the 30-day Brier score was 0.0886, which was highly consistent with the results of the training set, indicating that the model had no obvious overfitting.

#### 3.6.3. External validation.

A total of 317 SIC patients were included in the external validation cohort. Baseline characteristics of the external validation cohort are summarized in S4 Table in S1 File. Baseline characteristics between the derivation (MIMIC) and external validation cohorts were compared in S5 Table in S1 File, revealing significant differences in hypertension prevalence, vasopressor administration, and 30-day mortality, which may partially explain the slight reduction in discriminative performance observed in the validation set. The model achieved a C-index of 0.735 and an AUC of 0.761 (95% CI: 0.682–0.840) in the external validation cohort. Although it was slightly lower than that of the training set, it was still at a good level. To adjust for potential systematic bias, we performed Platt scaling recalibration in the external validation cohort. This common post-hoc calibration technique for clinical prediction models maps the Cox model’s linear predictors to interpretable absolute probabilities (0–1) through logistic regression fitting, and it improved the overall calibration of the model significantly. The 30-day calibration curve had no obvious deviation, and the 30-day Brier score was 0.1159, indicating excellent consistency between the predicted probability and the actual outcomes. Only due to the relatively limited sample size of the external cohort, the sample size corresponding to some probability intervals was insufficient, resulting in a slightly shortened coverage range of the calibration curve; the DCA curve maintained a positive net benefit within the threshold probability of 0–0.5, suggesting that the model may have good generalizability (S6 Fig in S1 File).

## 4. Discussion

Based on a cohort of 2531 SIC patients from the MIMIC-IV database and an independent external cohort from a Grade A tertiary hospital in Shanghai, this study systematically explored for the potential independent predictive value of high baseline RDW and rapidly ascending RDW dynamic trajectory for 30-day all-cause mortality in SIC patients, and developed a clinically practical nomogram model. This finding may supplement the gap in RDW prognostic research in the special population with SIC and also may overcome some limitations of static indicators through dynamic trajectory analysis, providing new biomarkers and convenient tools for SIC risk stratification.

This study confirmed that high baseline RDW is an independent risk factor for short-term mortality in SIC patients, which is consistent with the conclusions of relevant studies in general sepsis patients. For example, a meta-analysis by Wu et al. [32] showed that elevated RDW is significantly associated with increased short-term mortality in sepsis patients; Wang et al. [12] also pointed out that RDW can be used as a convenient prognostic biomarker for sepsis. This study, to our knowledge, first suggested the potential independence and dose-response relationship of this association in the special population with SIC, further expanding the clinical application scenario of RDW.

From a pathophysiological perspective, the association between RDW and SIC prognosis may originate from the interaction of multiple pathways. On the one hand, elevated RDW may reflect increased heterogeneity of red blood cell size, which will increase hemodynamic resistance and potentially promote platelet aggregation and microthrombus formation [15,16]; while SIC patients themselves have coagulation system activation, and the two may form a vicious circle, further aggravating organ hypoperfusion [33]. On the other hand, SIC patients are often accompanied by severe inflammatory response and oxidative stress [34]. Inflammatory response may directly inhibit bone marrow erythropoiesis and damages red blood cell membrane stability, while oxidative stress accelerates red blood cell aging; these changes may together lead to increased heterogeneity of red blood cell size [14], which may ultimately manifest as elevated RDW. In addition, elevated RDW is often accompanied by iron metabolism disorders and nutritional imbalances [35,36], which may further aggravate coagulation dysfunction in SIC patients and exacerbate tissue hypoxia and organ damage [37]. Clinically, patients in the Q3 group had higher SOFA scores, Charlson Comorbidity Index, CRRT utilization rate, and more significant abnormalities in liver, kidney, and coagulation indicators, which further suggested the association between high baseline RDW and disease severity as well as poor prognosis.

Moreover, the RDW dynamic trajectory constructed by LCGMM in this study more accurately reflects the disease progression trend. The 30-day mortality of patients with the rapidly ascending trajectory (Traj1) was significantly higher than that of the stable low-level type (Traj0), and this association remained stable after multivariable adjustment, indicating that dynamic changes in RDW are important signals of increased systemic stress and progressive deterioration of organ function. Compared with static baseline, dynamic trajectory has unique advantages: patients in the Traj1 group had more significant abnormalities in lactate and total bilirubin, higher CRRT utilization rate, and even higher mortality than the high baseline RDW group, indicating that dynamic trajectory can more sensitively identify high-risk groups with rapid disease deterioration. Previous research results in the field of sepsis provide important evidence for this finding: Gupta MK et al. [38] found that dynamic elevation of RDW is associated with increased 28-day mortality in patients with severe sepsis; Fan YW et al. [39] confirmed that RDW fluctuation and elevation rate are independent risk factors for sepsis-related disseminated intravascular coagulation (DIC) and death. However, this study attempted to achieve relatively more refined risk stratification through trajectory classification in the SIC population, and subgroup analysis confirmed that this predictive value has good robustness in most clinical scenarios such as mechanical ventilation, CRRT use, and diabetes, with no significant interaction, further verifying its reliability as a prognostic biomarker.

Notably, for clinical practitioners seeking to apply this approach in real-world settings, clarifying the practical manifestations of the two RDW pathways and the real-time identification of Traj1 patients is of great value. In clinical practice, the “stable low-level trajectory (Traj0)” typically presents as consistent RDW values (maintaining below the critical threshold) in repeated tests within 7 days after ICU admission, indicating relatively stable red blood cell metabolism and mild systemic stress response. In contrast, the “rapidly ascending trajectory (Traj1)” is characterized by a continuous upward trend in RDW (e.g., baseline RDW in the mid-range but increasing by >2% within 3–5 days, or baseline RDW already in the high range with further elevation). Clinicians can identify Traj1 patients through routine daily RDW testing: for SIC patients admitted to the ICU, dynamic monitoring of RDW levels every 1–2 days within the first week, combined with changes in lactate and organ function indicators (e.g., increasing TBIL, Cr), can promptly flag individuals at high risk of rapid deterioration. Such real-time identification allows for earlier optimization of treatment strategies (e.g., strengthening nutritional support, adjusting anti-inflammatory therapy, or intensifying organ function protection), which may improve clinical outcomes.

The nomogram constructed based on age, SOFA, lactate, VP, INR, and RDW showed good discrimination, calibration, and clinical utility through internal and external validation. It is important to note that the RDW trajectory classification was not included in the final nomogram of this study, a decision made after systematic variable selection and in line with the clinical positioning of the nomogram as a concise bedside predictive tool. Trajectory analysis successfully identified the rapidly ascending RDW pattern as a strong prognostic marker for poor outcomes in patients with SIC, which is also a core scientific finding of this study that reveals the prognostic significance of dynamic changes in RDW. However, the trajectory classification was highly correlated with baseline RDW, which already captures most of the prognostic information associated with dynamic RDW changes, and adding the trajectory classification to the multivariable model only resulted in a minimal improvement in the model’s predictive performance (the C-index slightly changed from 0.7998 to 0.7983, likelihood ratio test, *P* = 0.345). Therefore, to prioritize the simplicity, clinical interpretability and ease of use of the nomogram, we ultimately chose to retain baseline RDW instead of the trajectory classification, and all variables included in the nomogram are routine bedside test indicators available upon the patient’s ICU admission. Based on this, the nomogram enables early risk stratification and disease progression monitoring of SIC patients without additional medical costs, serving as a practical tool for clinicians to adjust treatment plans in a timely manner and optimize the allocation of medical resources.

This study has several limitations. The primary limitation is its retrospective design, which may introduce selection bias and unmeasured confounding factors affecting result robustness. For the trajectory analysis, the requirement of at least three RDW measurements within 7 days after ICU admission inherently excluded patients who died early or had insufficient tests, potentially leading to survivor bias. Although we have verified the stability of the results through a sensitivity analysis that excluded 31 patients who died within 72 hours after ICU admission, with the core conclusions remaining materially unaffected, as suggested by the reviewer, landmark analysis remains a more robust method to mitigate such bias in future studies. Additionally, some iron metabolism and nutrition-related indicators were excluded due to high missing rates [40], limiting the comprehensiveness of mechanistic analysis between RDW and SIC outcomes. Another limitation is the small external validation cohort (n = 317). Combined with the baseline differences between the derivation and validation cohorts, this limitation may compromise the statistical robustness of our findings, as well as the stability and generalizability of the nomogram in real-world clinical settings involving more heterogeneous patient populations. However, the application of recalibration helped mitigate this issue, yet challenges in generalizability persist. Insufficient sample representation in certain probability intervals narrows the calibration curve’s coverage and hinders full verification of predictive accuracy across all risk stratifications. Moreover, the study did not explore RDW’s predictive value stratified by ISTH SIC score, leaving unclear if its prognostic utility varies with SIC severity.

To address these, future studies should conduct multicenter, prospective trials with larger sample sizes to validate the nomogram in diverse settings. Basic experiments are needed to clarify the molecular mechanisms between RDW and SIC-related coagulation dysfunction. Targeted interventions for high-risk populations such as optimizing nutrition or modulating inflammation should also be explored. Expanding external validation sample sizes will better assess the model’s calibration across subgroups and clinical scenarios, enhancing its clinical reliability. These efforts aim to strengthen the theoretical basis for personalized SIC management.

## 5. Conclusion

High baseline RDW and rapidly ascending RDW dynamic trajectory may be independent risk factors for 30-day all-cause mortality in SIC patients, and the dynamic trajectory may have relatively more sensitive predictive value for identifying high-risk patients with rapid disease progression. The nomogram constructed based on RDW combined with relevant indicators has been validated internally and externally, and may be conveniently and accurately used for potential risk stratification and prognostic evaluation of SIC patients.

## Supporting information

S1 File Supplementary tables and figures. (DOCX)

## Abbreviations

RDW: Red Blood Cell Distribution Width
SIC: Sepsis-Induced Coagulopathy
MIMIC-IV: Medical Information Mart for Intensive Care IV
LCGMM: Latent Class Growth Mixture Model
KM: Kaplan-Meier
RCS: Restricted Cubic Spline
HR: Hazard Ratio
CI: Confidence Interval
ISTH: International Society on Thrombosis and Haemostasis
SOFA: Sequential Organ Failure Assessment
MIT: Massachusetts Institute of Technology
BIDMC: Beth Israel Deaconess Medical Center
INR: International Normalized Ratio
PLT: Platelet
RBC: Red Blood Cell
SQL: Structured Query Language
DM: Diabetes Mellitus
HTN: Hypertension
CKD: Chronic Kidney Disease
TBIL: Total Bilirubin
ALB: Albumin
CRP: C-Reactive Protein
Lac: Lactate
Hb: Hemoglobin
HCT: Hematocrit
APTT: Activated Partial Thromboplastin Time
Cr: Creatinine
MV: Mechanical Ventilation
CRRT: Continuous Renal Replacement Therapy
VP: Vasopressors
FFP: Fresh Frozen Plasma
IQR: Interquartile Range
VIF: Variance Inflation Factor
AIC: Akaike Information Criterion
BIC: Bayesian Information Criteria
SABIC: Sample-Adjusted Bayesian Information Criterion
DIC: Disseminated Intravascular Coagulation
AUC: Area Under the Curve
DCA: Decision Curve Analysis
