# Supplementary material for: Baseline RDW combined with dynamic trajectory: Predictive value for 30-day all-cause mortality in patients with sepsis-induced coagulopathy and development of a nomogram
Source: PLoS One. 2026 Apr 27;21(4):e0348149. doi: 10.1371/journal.pone.0348149 (PMC13120281; doi:10.1371/journal.pone.0348149)
Supplement: S1 File — (DOCX) [file pone.0348149.s001.docx]

**S1 Table. Missing rates and handling of predictor variables**

| **Variable Name** | **Missing Rates (%)** |
| --- | --- |
| RDW | 0 |
| Age | 0 |
| Gender | 0 |
| SOFA | 0 |
| Charlson | 0 |
| Hemoglobin | 0 |
| RBC | 0 |
| Hematocrit | 0.1 |
| TBIL | 29.8 |
| APTT | 0.5 |
| INR | 0 |
| PLT | 0 |
| Lac | 7.2 |
| ALB | 42.3 |
| CRP | 91.0 |
| Cr | 0 |
| D-Dimer | 97.2 |
| Functional Fibrinogen | 37.3 |
| Folic Acid | 94.5 |
| Vitamin B12 | 92.0 |
| Ferritin | 79.9 |
| Transferrin | 81.4 |

Variables with a missing rate > 30% were excluded, including albumin (ALB), C-reactive protein (CRP), D-Dimer, functional fibrinogen, folic acid, vitamin B12, ferritin, and transferrin.

RDW: Red blood cell distribution width; SOFA: Sequential Organ Failure Assessment; RBC: Red blood cell count; TBIL: Total bilirubin; APTT: Activated partial thromboplastin time; INR: International normalized ratio; PLT: Platelet; Lac: Lactate; ALB: Albumin; CRP: C-reactive protein; Cr: Creatinine.

**S2 Table. Variance inflation factors (VIFs) for all included predictor variables**

| **Variable Name** | **VIF (Baseline RDW)** | **VIF (RDW Trajectory )** |
| --- | --- | --- |
| RDW | 1.0977 | 1.0875 |
| Age | 1.1281 | 1.1271 |
| Gender | 1.0279 | 1.0342 |
| SOFA | 1.1902 | 1.1823 |
| VP | 1.0669 | 1.0631 |
| Therapeutic heparin | 1.0556 | 1.0549 |
| FFP | 1.0383 | 1.0310 |
| CRRT | 1.1366 | 1.1259 |
| MV | 1.0175 | 1.0173 |
| HTN | 1.3004 | 1.2711 |
| CKD | 1.3810 | 1.3577 |
| DM | 1.0424 | 1.0495 |
| RBC | 1.0888 | 1.1041 |

VIF: Variance inflation factor; VP: Vasopressors; FFP: Fresh frozen plasma; CRRT: Continuous renal replacement therapy; MV: Mechanical ventilation; HTN: Hypertension; CKD: Chronic kidney disease; DM: Diabetes mellitus.

**S3 Table.** **Table of non-zero lambda coefficients in LASSO regression**

| **Coef Name** | **Lambda Coefficients** |
| --- | --- |
| Age | 0.2597 |
| SOFA | 0.3918 |
| VP | -0.9316 |
| RDW | 0.2574 |
| INR | 0.0117 |
| Lac | 0.0894 |

The optimal lambda value is 0.012, which was determined based on the minimum mean squared error from 10-fold cross-validation combined with the one-standard-error rule.

SOFA: Sequential Organ Failure Assessment; VP: Vasopressors; RDW: Red blood cell distribution width; INR: International normalized ratio; Lac: Lactate.

**S4 Table. Baseline characteristics of patients with sepsis-induced coagulopathy in the external validation cohort**

|  | **Overall**  **(N = 317)** | **30-Day Survivors**  **(n = 265)** | **30-Day Non-Survivors**  **(n = 52)** | ***P*** |
| --- | --- | --- | --- | --- |
| **Demographics​** |  |  |  |  |
| Age (years) | 69.0 (20.0) | 68.0 (19.0) | 72.0 (18.0) | 0.068 |
| Gender (Male, n%)​ | 204 (64.4) | 168 (63.4) | 36 (69.2) | 0.432 |
| **Disease Severity Scores** |  |  |  |  |
| SOFA | 7.0 (4.0) | 7.0 (4.0) | 10.0 (4.3) | <0.001 |
| **Laboratory Markers** |  |  |  |  |
| RDW (%) | 14.8 (2.4) | 14.7 (2.3) | 15.8 (2.6) | 0.006 |
| INR | 1.6 (0.5) | 1.6 (0.4) | 1.9 (0.7) | 0.086 |
| PLT (×10⁹/L) | 130.0 (92.0) | 132.0 (90.0) | 118.0 (88.0) | 0.275 |
| Lac (mmol/L) | 2.0 (1.6) | 2.0 (1.5) | 1.9 (1.7) | 0.994 |
| **First-day Treatments** |  |  |  |  |
| Therapeutic heparin (n%)​ | 35 (11.0) | 29 (10.9) | 6 (11.5) | 0.893 |
| MV (n%)​ | 257 (81.1) | 215 (81.1) | 42 (80.8) | 0.957 |
| CRRT (n%)​ | 11 (3.5) | 8 (3.0) | 3 (5.8) | 0.365 |
| VP (n%)​​ | 117 (36.9) | 105 (39.6) | 12 (23.1) | 0.021 |
| FFP (n%)​ | 46 (14.5) | 38 (14.3) | 8 (15.4) | 0.834 |
| **Comorbidities** |  |  |  |  |
| DM (n%)​ | 103 (32.5) | 85 (32.1) | 18 (34.6) | 0.731 |
| HTN (n%)​ | 156 (49.2) | 129 (48.7) | 27 (51.9) | 0.654 |
| CKD (n%)​ | 52 (16.4) | 43 (16.2) | 9 (17.3) | 0.847 |

Continuous variables are presented as median (IQR), and categorical variables are expressed as n (%).

SOFA: Sequential Organ Failure Assessment; RDW: Red blood cell distribution width; INR: International normalized ratio; PLT: Platelet; Lac: Lactate; MV: Mechanical ventilation; CRRT: Continuous renal replacement therapy; VP: Vasopressors; FFP: Fresh frozen plasma; DM: Diabetes mellitus; HTN: Hypertension; CKD: Chronic kidney disease.

**S5 Table. Baseline characteristics of the derivation (MIMIC) and external validation cohorts**

|  | **MIMIC cohort**  **(n=2531)** | **External validation cohort (n=317)** | ***P*** |
| --- | --- | --- | --- |
| **Demographics​** |  |  |  |
| Age (years) | 69.0 (20.0) | 69.0 (20.0) | 0.892 |
| Gender (Male, n%)​ | 1665 (65.8) | 204 (64.4) | 0.614 |
| **Disease Severity Scores** |  |  |  |
| SOFA | 7.0 (4.0) | 7.0 (4.0) | 0.947 |
| **Laboratory Markers** |  |  |  |
| RDW (%) | 14.5 (1.9) | 14.8 (2.4) | 0.012 |
| INR | 1.6 (0.4) | 1.6 (0.5) | 0.783 |
| PLT (×10⁹/L) | 129.0 (80.0) | 130.0 (92.0) | 0.659 |
| Lac (mmol/L) | 2.0 (1.6) | 2.0 (1.6) | 0.911 |
| **First-day Treatments** |  |  |  |
| Therapeutic heparin (n%)​ | 233 (9.2) | 35 (11.0) | 0.302 |
| MV (n%)​ | 2053 (81.1) | 257 (81.1) | 0.987 |
| CRRT (n%)​ | 61 (2.4) | 11 (3.5) | 0.287 |
| VP (n%)​​ | 1372 (54.2) | 117 (36.9) | <0.001 |
| FFP (n%)​ | 124 (4.9) | 46 (14.5) | <0.001 |
| **Comorbidities** |  |  |  |
| DM (n%)​ | 825 (32.6) | 103 (32.5) | 0.971 |
| HTN (n%)​ | 990 (39.1) | 156 (49.2) | 0.001 |
| CKD (n%)​ | 564 (22.3) | 52 (16.4) | 0.018 |
| **Clinical outcome** |  |  |  |
| 30-day mortality, n (%) | 319 (12.6) | 52 (16.4) | 0.049 |

**S6 Table. Multivariable Cox regression coefficients of the nomogram model**

| **Variable** | **Coefficient** | **Hazard Ratio** | **Standard Error** | ***Z*** | ***P*** |
| --- | --- | --- | --- | --- | --- |
| Age | 0.7017 | 2.0171 | 0.1272 | 5.5145 | <0.001 |
| SOFA | 0.1625 | 1.1765 | 0.0168 | 9.6528 | <0.001 |
| VP | -1.3502 | 0.2592 | 0.1396 | -9.6714 | <0.001 |
| RDW | 0.1471 | 1.1585 | 0.0185 | 7.9420 | <0.001 |
| INR | 0.0677 | 1.0700 | 0.0332 | 2.0407 | 0.041 |
| Lac | 0.0561 | 1.0577 | 0.0185 | 3.0387 | 0.002 |

**
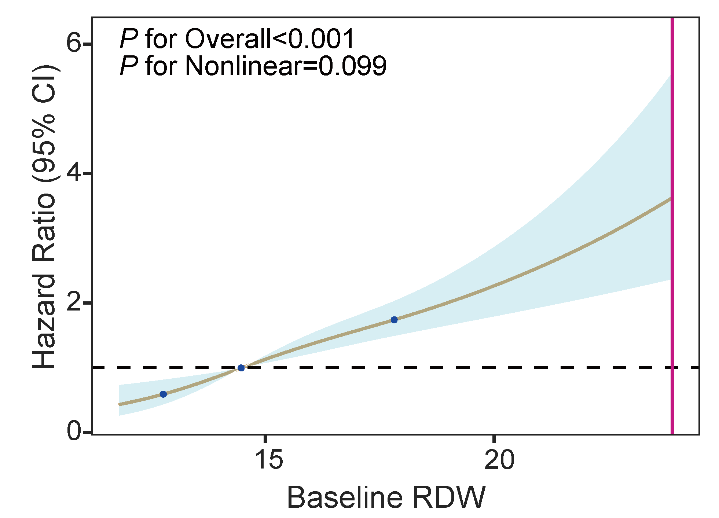

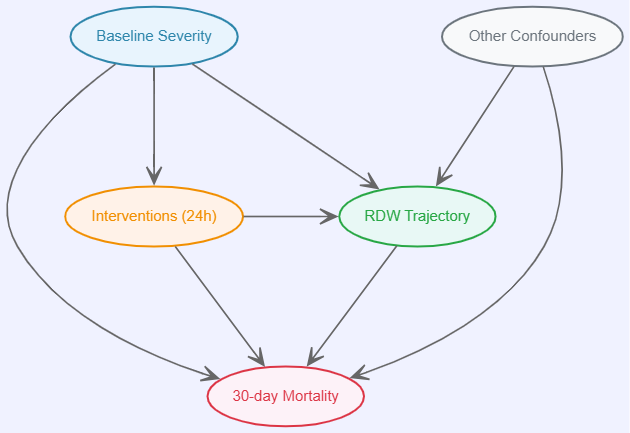
S1 Fig. Directed acyclic graph (DAG) for causal inference in this study.** DAG demonstrating causal relationships among baseline disease severity, early interventions (e.g., CRRT, vasopressor therapy), other confounders (e.g., age, comorbidities), baseline and longitudinal trajectory of red blood cell distribution width (RDW), and 30-day mortality.

**S2 Fig.** **Multivariable-adjusted restricted cubic spline (RCS) of baseline RDW and 30-day all-cause mortality.
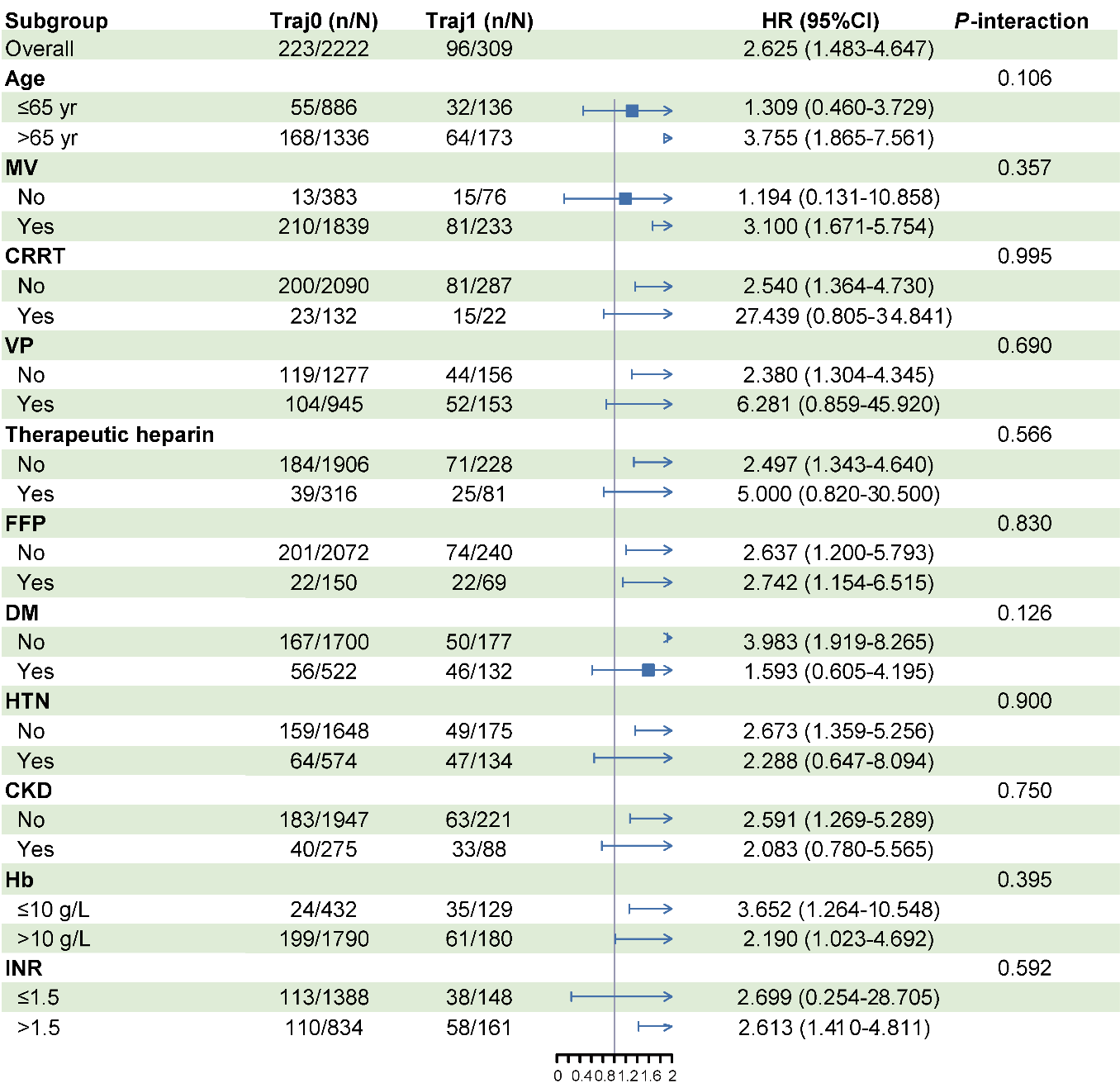
**

**S3
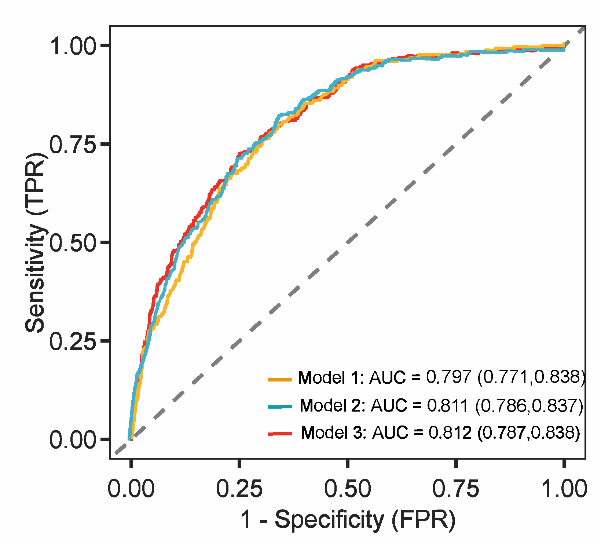
 Fig.** **Forest plot of the association between RDW trajectories and 30-day all-cause mortality in subgroups.**

**S4 Fig.** **ROC curves for predicting 30-day all-cause mortality in patients with sepsis-induced coagulopathy.** Model 1 (base clinical model); Model 2 (clinical + baseline RDW); Model 3 (clinical + RDW trajectory)**
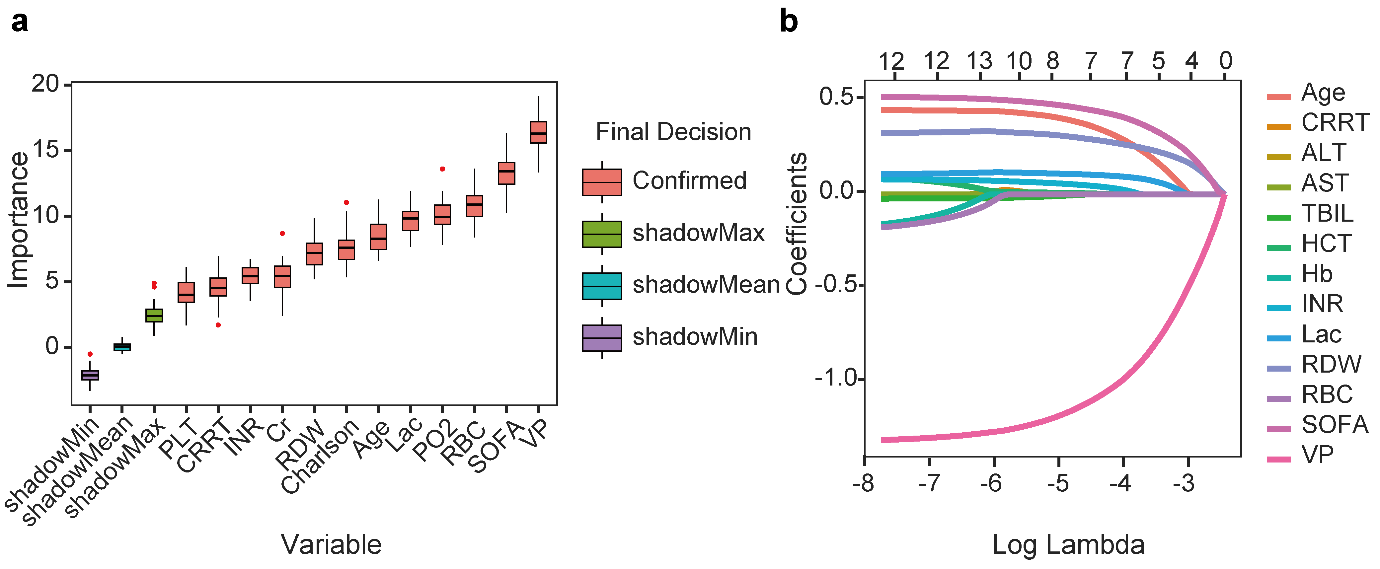
**

**S5 Fig.** **Selection and evaluation of predictive features.** A. Variable importance ranking evaluated based on the Boruta algorithm; B. Coefficient path plot of LASSO regression.
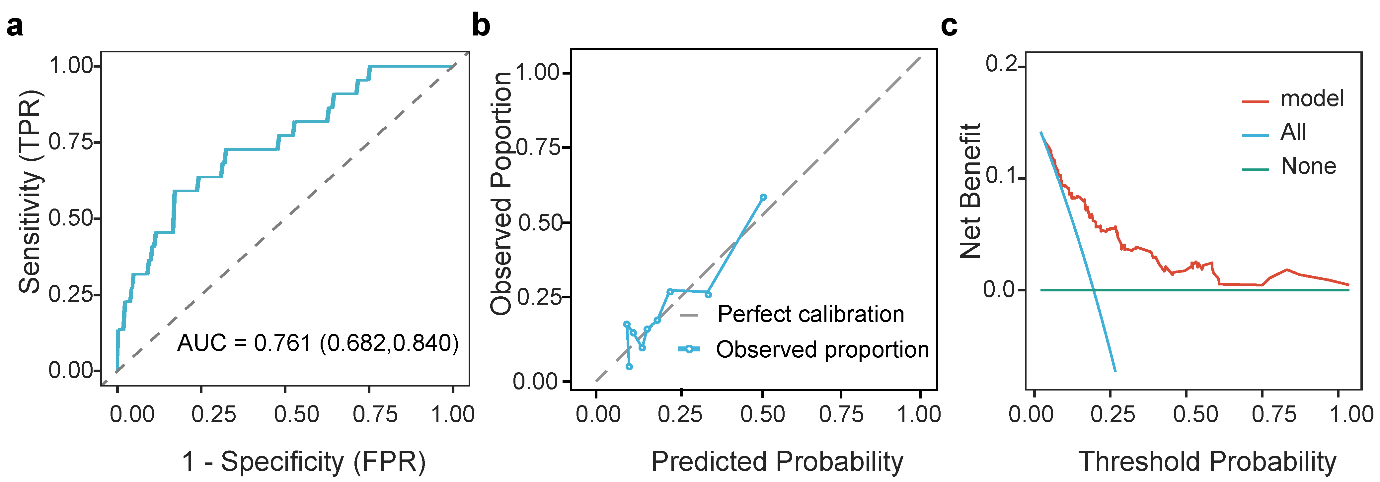


**S6 Fig. The performance of the nomogram in the external validation set.** A. ROC Curve; B. Calibration Curve; C. Decision Curve Analysis.
